# Supplementary material for: Severe acute respiratory syndrome coronavirus (SARS-CoV-2) is not detected in the vagina: A prospective study
Source: PLoS One. 2021 Sep 30;16(9):e0253072. doi: 10.1371/journal.pone.0253072 (PMC8483336; doi:10.1371/journal.pone.0253072)
Supplement: S1 Protocol — (DOCX) [file pone.0253072.s002.docx]

**ACIBADEM ÜNİVERSİTESİ VE ACIBADEM SAĞLIK KURULUŞLARI**

**TIBBİ ARAŞTIRMA İZİN VE ONAY BAŞVURU FORMU**

**Bu bölümü boş bırakın. İlgili komisyonca doldurulacaktır.**

| **Toplantı tarihi** |  |
| --- | --- |
| **Karar numarası** |  |

|  |  |
| --- | --- |
| **Değişiklik** |  |
| **Ret** |  |

**1. GENEL BİLGİLER**

**________________________________________________________________**

**A) Tıbbi Araştırmanın Başlığı:**

**Covid-19 tanısı ile pnomoni tedavisi alan hastalarda vajinal sürüntüde Covid-19 pozitifliğinin bakılması**

**__________________________________________________________________________**

**B) Araştırma Sorumlusu (ları) (Unvanı, Adı ve Soyadı, Birimi, Telefon, e-mail):**

Dr.Öğr.Üyesi Özgüç Takmaz- Acıbadem MAA Üniversitesi Kadın Hastalıkları ve Doğum AD- 02123044497, [ozguc.takmaz@acibadem.edu.tr](mailto:ozguc.takmaz@acibadem.edu.tr)

**C) Yardımcı Araştırıcı (lar) (Unvanı, Adı ve Soyadı, Birimi, Telefon, e-mail):**

Prof. Dr. Serap Gençer – Acıbadem MAA Üniversitesi Enfeksiyon Hastalıkları – 05422451702 – serap.gencer@acibadem.com

Dr. Öğretim Üyesi Neval Yurttutan Uyar – Acıbadem MAA Üniversitesi Tıbbi Mikrobiyoloji AD – 05306143181 – neval.uyar@acibademlabmed.com.tr

Arş. Gr. Dr. Eren KAYA – Acıbadem MAA Üniversitesi Kadın Hastalıkları ve Doğum AD – 02123044497, erenkaya@acibadem.com

**-----------------------------------------------------------------------------------------------------------------**

**D) Araştırma Yardımcısı (ları) (Unvanı, Adı ve Soyadı, Birimi, Telefon, e-mail):**

**E) Tıbbi Araştırmanın Yürütüleceği Birimler (Anabilim Dalı, Klinik, Poliklinik, Laboratuvar ve benzeri).**

Çalışma Acıbadem MAA Üniversitesi Kadın Hastalıkları ve Doğum Anabilim dalında yürütülecektir. Çalışmanın yürütüleceği klinikler Acıbadem Maslak Hastanesi pandemi servisleri ve Acıbadem Merkez Laboratuarıdır. (LABMED)

**2. TIBBİ ARAŞTIRMANIN AMACI, BİLİMSEL DAYANAĞI VE YÖNTEMLER**

- - 1. **Tıbbi Araştırmanın Amacı (***100 kelimeyi geçmeyecek şekilde belirtiniz)***:**

2019 yılı Aralık ayında Çin'in Wuhan kentinde tanımlanan SARS-CoV dünya çapında etkisinini gösteren bir pandemi haline gelmiştir. Bu virüsün tedavisi ve önlenmesi için yapılan çalışmaların yanında bulaş yolları ve yenidoğan, gebe ve ileri yaşta hastalar gibi riskli popülasyondaki etkisi araştırılmaya devam etmektedir. Önceki yıllarda yapılan çalışmalarda üreme çağındaki kadın popülasyonda coronavirüs türlerinin vajinal florada saptandığı gösterilmiş olmasına rağmen, covid-19 virüsünün neden olduğu SARS-CoV vakalarında bu konuyla ilgili sınırlı sayıda hastada yapılmış bir çalışmada vajinal florada Covid-19 virüsü bakılmış ancak saptanmamıştır. Bu çalışmadaki amacımız, covid-19 tanısı almış ve radyolojik olarak viral pnömoniyle uyumlu bulunan kadın hastaların tedaviye başlanmadan önce yapılan vajinal sürüntü örneklerinde covid-19 virüsü varlığının araştırılması ve Covid-19 un cinsel yolla veya vajinal doğum ile bulaş riskinin değerlendirilmesidir. Ayrıca, vajinal sürüntüde Covid-19 pozitifliği saptanan hastalarda tedavi sonrası tekrar alinan vajinal örneklerde Covid-19 pozitifliğinin değerledirilmesidır.

- - 1. **Tıbbi Araştırmanın Bilimsel Dayanağı ve Geçerliliği** *[Bu bölümde tıbbi araştırmanın neden yapılmak istendiği, dayanağı ve geçerliliğini konu ile ilgili kaynaklar göstererek 300 kelimeyi geçmeyecek şekilde belirtiniz]*:

Vajinal akıntıda Covid-19 pozitifliği araştırılması ile ilgili günümüzde 10 hasta içeren bir yayın bulunmaktadır (1). Bu yayında da Covid-19 pnomonisi ile tedavi edilen kadın hastaların vajinal sürüntülerinde virüs saptanmamıştır. Covid-19 virüsüyle ilgili gebelerde ve cinsel yolla bulaş konusunda da sınırlı sayıda yayın literatüre geçmiş olup, derlemelerde bu alanda yeni çalışmaların yapılması gerekliliği belirtilmiştir (2,3,6). Gebe popülasyonda covid-19 hastalığı saptanan tüm vakalarda 3. trimesterde gözlenmiş olup bu da doğumun zamanlaması ve doğum yöntemiyle ilgili soruları gündeme getirmiştir (7). Cinsel yolla bulaş konusunda yapılan 35 hastalık bir çalışmada bu virüsün cinsel yolla bulaşı gösterilememiş ancak asemptomatik hastaların sayısının fazlalığı ve virüsün bulaş hızının çok yüksek olması nedeniyle bu konuda daha fazla çalışmaya ihtiyaç duyulduğu belirtilmiştir (4). Covid-19 nedeni ile viral pnomoni tanısı alan, gebe olmayan veya gebe olan kadın hastaların vajinal sürüntülerinde Covid-19 bakılarak, bu hastalığın cinsel yolla bulaşma ihtimalini ve vajinal yolla doğum sırasında yeni doğana bulaşma ihtimalini değerlendirmek, hem hastalığın yayılmasını engellemek hem de yenidoğanları bu enfeksiyondan korumak için önemli bilgiler edinmemizi sağlayacaktır.

Referanslar

1.Lin Qiu, Xia Liu, Meng Xiao, Jing Xie, Wei Cao, Zhengyin Liu, Abraham Morse, Yuhua Xie, Taisheng Li, Lan Zhu, *SARS-CoV-2 is not detectable in the vaginal fluid of women with severe COVID-19 infection*, Clinical Infectious Diseases, , ciaa375

2. Archives of Pathology & Laboratory Medicine Online. 2020. *An Analysis Of 38 Pregnant Women With COVID-19, Their Newborn Infants, And Maternal-Fetal Transmission Of SARS-Cov-2: Maternal Coronavirus Infections And Pregnancy Outcomes*. [online] Available at: <https://www.archivesofpathology.org/doi/abs/10.5858/arpa.2020-0901-SA> [Accessed 9 April 2020].

3. Chen, H., Guo, J., Wang, C., Luo, F., Yu, X., Zhang, W., Li, J., Zhao, D., Xu, D., Gong, Q., Liao, J., Yang, H., Hou, W. and Zhang, Y., 2020. *Clinical Characteristics And Intrauterine Vertical Transmission Potential Of COVID-19 Infection In Nine Pregnant Women: A Retrospective Review Of Medical Records*.

4. Cui, P., Chen, Z., Wang, T., Dai, J., Zhang, J., Ding, T., Jiang, J., Liu, J., Zhang, C., Shan, W., Wang, S., Rong, Y., Chang, J., Miao, X., Ma, X. and Wang, S., 2020. *Clinical Features And Sexual Transmission Potential Of SARS-Cov-2 Infected Female Patients: A Descriptive Study In Wuhan, China*.

5. Qiao, J., 2020. *What Are The Risks Of COVID-19 Infection In Pregnant Women?*.

6. Schwartz, D. and Graham, A., 2020. *Potential Maternal And Infant Outcomes From Coronavirus 2019-Ncov (SARS-Cov-2) Infecting Pregnant Women: Lessons From SARS, MERS, And Other Human Coronavirus Infections*.

7. Taylor & Francis. 2020. *Vertical Transmission Of Coronavirus Disease 19 (COVID-19) From Infected Pregnant Mothers To Neonates: A Review*. [online] Available at: <https://www.tandfonline.com/doi/full/10.1080/15513815.2020.1747120> [Accessed 9 April 2020].

- - 1. **Araştırmanın Yöntemi ve Uygulanacak işlemler** *[Bu bölümde hasta ve sağlıklı katılımcı bilgileri, materyallerin nasıl ve ne sıklıkla toplanacağı, ölçülecek parametreler, gibi özellikler ayrıntılı şekilde belirtilmelidir]*:

Çalışmada Acıbadem Maslak Hastanesi acil servisinde değerlendirilen ve klinik bulguları Covid-19 ile uyumlu olan hastalardan radyolojik olarak viral pnömoni düşülen kadın hastalar servise yatırılarak tedavi başlamadan nazofarengeal sürüntü ile eş zamanlı vajinal sürüntü de alınarak vajinal Covid-19 araştırılacaktır. Covid-19 sürüntü örnekleri Acıbadem Merkez Laboratuarında çalışılacaktır. Nazofarengeal sürüntüleri covid (+) olan ile klinik ve radyolojik olarak covid-19 ile uyumlu olan ve nazofarengeal sürüntüsü (-) olan hastalarda vajinal sürüntüdeki covid-19 pozitifliği araştırılacaktır. Vajinal Covid-19 pozitifliği saptanan hastalarda tedavi sonrası tekrar vajinal Covid-19 testi yapılarak vajinal Covid-19 negatifleşmesi değerlendirilecektir.

- - 1. **Gönüllülerin Araştırmaya Alınma ve Dışlanma Kriterleri [***maddeleyerek yazınız]* :

Çalışmaya ilk başvuruda klinik ve radyolojik olarak covid-19 düşünülen 18 yaşını doldurmuş tüm kadın hastalar dahil edilecektir.

Daha önceden covid-19 nedeniyle tedavi almış hastalar çalışma dışı tutulacaktır.

- - 1. **Araştırmaya Alınacak Gönüllü Sayısı:**

Araştırmamıza kriterlere uygun 60 gönüllü hasta alınacaktır.

- - 1. **Araştırmanın Süresi**:

Araştırmanın 1 ay içinde sonuçlanması planlanmaktadır.

- - 1. **Araştırma Desteği** *[araştırma harcamalarının nasıl ve nereden karşılanacağı, varsa destek veren kuruluşlar ve destek miktarı]:*

Covid-19 testlerinin ülkemizde belirli merkezlerde ve ücretsiz uygulanması nedeniyle araştırmamızın ek bir harcama bütçesi bulunmamaktadır.

**3. ARAŞTIRMANIN NİTELENDİRİLMESİ**

**3.1 Size göre araştırmanız hangisine (Yönergenin 6. maddesine göre) uymaktadır** *[Lütfen işaretleyiniz]*

**A) Klinik Araştırmalar Hakkında Yönetmelik kapsamında yer alan araştırmalar ( )**

( ) İlaç Araştırmaları

( ) Ticari Olmayan İlaç Araştırmaları

( ) Tıbbi Cihazlarla Yapılan Araştırmalar

( ) Yeni Cerrahi Yöntem Kullanılarak Yapılacak Araştırmalar

( ) İlaç Biyoyararlanım ve Biyoeşdeğerlik Çalışmaları

( x ) Tanı ve Tarama Testlerinin Araştırılması

**B) Klinik Araştırmalar Hakkında Yönetmelik kapsamında yer almayan tıbbi araştırmalar ( X )**

( ) Gözlemsel İlaç Araştırmaları

( ) Gözlemsel Epidemiyolojik Araştırmalar

( ) Anket, Sorgulama ve Görüşme Şeklinde Tıbbi Araştırmalar

( x ) İnsan Biyolojik Materyallerinin Kullanımını İçeren Tıbbi Araştırmalar

( x ) Tıbbi Kayıtlar ve Hasta Bilgileri Üzerinde Yapılan Araştırmalar

( ) Nitel Araştırmalar

**3.2 Size göre araştırmanız hangi risk (Yönergenin 7. maddesine göre) değerlendirmesine uymaktadır** *[Lütfen işaretleyiniz]*

( X) Risk İçermeyen Tıbbi Araştırmalar

( ) Minimal Risk İçeren Tıbbi Araştırmalar^[[1]](#footnote-1)^*

( ) Ek Risk İçeren Tıbbi Araştırmalar*

( ) Belirsiz Risk İçeren Tıbbi Araştırmalar

**3.3 Araştırmanız hangi insan gurubu (Yönergenin 8. maddesine göre) üzerinde yürütülecektir** *[Lütfen işaretleyiniz]*

( ) Hastalar Üzerinde [Tedavi ile beraber ( ) Tedaviden bağımsız (X)]

( ) Sağlıklı İnsanlar Üzerinde

( ) Korunmasız ve Zarara Açık Gruplar Üzerinde

( ) Bebekler ve çocuklar

( ) Gebeler, lohusalar ve emziren anneler

( ) Öğrenme yetisi sınırlı olanlar

( ) Ağır ve kronik hastalar

( ) Acil hastalar

( ) Komadaki hastalar

( ) Yoğun bakım hastaları

( ) Psikiyatrik hastalar

( ) Alzheimer hastaları ve benzeri türden bunaması olan hastalar

( ) Umutsuz ve çaresiz hastalar

( ) Mahkûmlar

( ) Askerler

( ) Öğrenciler ve sağlık personeli

( ) Kaza geçirmiş kişiler

( ) Şiddete uğramış kişiler

( ) Afetzedeler

**4. AYDINLATILMIŞ ONAM FORMU** *[araştırmanın özelliğine göre Yönergenin 29. maddesindeki hususları kapsayacak şekilde hazırlayınız*

Sizi Dr.Öğr.Üyesı Özgüç Takmaz tarafından yürütülen “**Covid-19 tanısı alan hastada vajinal florada coronavirus saptanması ve covid-19 vertikal bulaş riski değerlendirilmesi**.” başlıklı araştırmaya davet ediyoruz. Bu arastirmanin amacı solunum sistemi ve boğazda tesbit edilen covid-19 virüsünün vajinal florada bulunup bulunmadığının araştırılmasıdır. Bu amacla tedaviniz öncesinde, eğer izin verirseniz, covid-19 şüpheli hastalarda yapılan rutin bir işlem olan ağız ve burun boşluğunuzdan alınan sürüntü örneğinin bir benzerini vajinal bölgeden alarak covid-19 virüsünün vajinal salgılarda/akıntılarda varlığını araştıracağız. Yapilacak olan örnekleme ve sonucu size yapılacak olan tedaviyi hiçbir şekilde etkilemeyecektir. Bu tetkik işlemleri için sizden ya da sigorta kurumunuzdan kesinlikle ek bir ucret talep edilmeyecektir. Ayrıca, vajinal sürüntü alınması işlemi cinsel aktif olmayan kadınlarda kızlık zarına herhangi bir zarar vermeyecektir.

Araştırmaya sizin dışınızda tahminen 59 kişi daha katılacaktır. Bu çalışmaya katılmak tamamen **gönüllülük** esasına dayanmaktadır. Bu formu okuyup onaylamanız, araştırmaya katılmayı kabul ettiğiniz anlamına gelecektir. Ancak, çalışmaya katılmama veya katıldıktan sonra herhangi bir anda çalışmayı bırakma hakkına da sahipsiniz. Bu çalışmadan elde edilecek bilgiler tamamen araştırma amacı ile kullanılacak olup kişisel bilgileriniz **gizli tutulacaktır**; ancak verileriniz yayın amacı ile kullanılabilir. Eğer araştırmanın amacı ile ilgili verilen bu bilgiler dışında şimdi veya sonra daha fazla bilgiye ihtiyaç duyarsanız araştırmacıya şimdi sorabilir veya ‘ozguc.takmaz@acibadem.com.tr’ e-posta adresi ve 02123044497 numaralı telefondan ulaşabilirsiniz. Araştırma tamamlandığında genel/size özel sonuçların sizinle paylaşılmasını istiyorsanız lütfen araştırmacıya iletiniz.

**Uygulanacak İşlemler:** Servise yatış işleminiz tamamlandıktan sonra rutin olarak yapılan ağız ve burun boşluğunuzdan alınan sürüntü örneğiyle aynı gün içinde, henüz tedaviye başlanmadan sizden bir vajinal sürüntü örneği alınacaktır. Tedaviniz tamamen ağız ve burun boşluğunuzdan alınan sürüntüye göre planlanacak olup araştırma amaçlı yapılan sürüntünün sonuçlarını dilerseniz sonrasında sizinle paylaşabiliriz. Eğer tedavi öncesi vajinal Covid-19 testiniz pozitif (var) çıktı ise, tedavi sonrası vajinal Covid-19 testiniz tekrarlanacaktır. Bu çalışma ile covid-19 hastalığının cinsel yolla bulaşını ve gebe populasyonda doğum sırasında bulaş riskini araştıracağız. Dilerseniz araştırma sonuçları yayımlandığında makalenin bir kopyasını size iletebiliriz.

Yukarıda yer alan ve araştırmadan önce katılımcıya verilmesi gereken bilgileri okudum ve katılmam istenen çalışmanın kapsamını ve amacını, gönüllü olarak üzerime düşen sorumlulukları anladım. Çalışma hakkında yazılı ve sözlü açıklama aşağıda adı belirtilen araştırmacı/araştırmacılar tarafından yapıldı. Bana, çalışmanın muhtemel riskleri ve faydaları sözlü olarak da anlatıldı. Kişisel bilgilerimin özenle korunacağı konusunda yeterli güven verildi.

Bu koşullarda söz konusu araştırmaya kendi isteğimle, hiçbir baskı ve telkin olmaksızın katılmayı kabul ediyorum.

**Katılımcının :**

Adı-Soyadı:.....................................................................................................................................

İmzası: e-posta: Telefon:

Velayet veya Vesayet Altında Bulunanlar İçin;

Veli veya Vasisinin

Adı-Soyadı:.....................................................................................................................................

İmzası:

**Araştırmacının**

Adı-Soyadı: Dr. Öğr.Üyesi. Özgüç Takmaz

*İmzası:*

1. * Riskleri tek tek belirtiniz ve bunlara aydınlatılmış onam formunda yer veriniz. [↑](#footnote-ref-1)
