# Supplementary material for: Severe acute respiratory syndrome coronavirus (SARS-CoV-2) is not detected in the vagina: A prospective study
Source: PLoS One. 2021 Sep 30;16(9):e0253072. doi: 10.1371/journal.pone.0253072 (PMC8483336; doi:10.1371/journal.pone.0253072)
Supplement: S2 Protocol — (DOCX) [file pone.0253072.s003.docx]

ACIBADEM UNIVERSITY AND ACIBADEM HEALTH INSTITUTIONS

MEDICAL RESEARCH PERMISSION AND APPROVAL APPLICATION FORM

1. GENERAL INFORMATION
2. Examination of Covid-19 positivity in vaginal swab in patients who received pneumonia treatment with the diagnosis of Covid-19
3. Research Supervisor (s) (Title, Name and Surname, Unit, Phone, e-mail):

Assistant Professor Özgüç Takmaz- Acıbadem MAA University Department of Obstetrics and Gynecology- 02123044497, [ozguc.takmaz@acibadem.edu.tr](mailto:ozguc.takmaz@acibadem.edu.tr)

C) Assistant Researcher (s) (Title, Name and Surname, Unit, Phone, e-mail):

Prof. Dr. Serap Gençer - Acıbadem MAA University Infectious Diseases - 05422451702 - serap.gencer@acibadem.com

Dr. Faculty Member Neval Yurttutan Uyar - Acıbadem MAA University Department of Medical Microbiology - 05306143181 - neval.uyar@acibademlabmed.com.tr

Res. Gr. Dr. Eren KAYA - Acıbadem MAA University Department of Obstetrics and Gynecology - 02123044497, [erenkaya@acibadem.com](mailto:erenkaya@acibadem.com)

1. Research Assistant (s) (Title, Name and Surname, Unit, Phone, e-mail):
2. Units where Medical Research will be conducted (Department, Clinic, Polyclinic, Laboratory and so on).

The study will be carried out in Acıbadem MAA University, Department of Obstetrics and Gynecology. The clinics where the study will be conducted are Acıbadem Maslak Hospital pandemic services and Acıbadem Central Laboratory. (LABMED)

2. AIM OF MEDICAL RESEARCH, SCIENTIFIC BASIS AND METHODS

A) Purpose of Medical Research (Include not exceeding 100 words):

SARS-CoV, which was firstly defined in Wuhan, China in December 2019, has become a pandemic showing its impact worldwide. In addition to the studies conducted for the treatment and prevention of this virus, the transmission routes and its effect on the risky population such as newborns, pregnant and elderly patients continue to be investigated. Although it has been shown in previous studies that coronavirus species are detected in the vaginal flora in the female population of reproductive age in a limited number of patients. On this issue, in SARS-CoV cases caused by the covid-19 virus, Covid-19 virus was examined in the vaginal flora but was not detected. Our aim in this study is to investigate the presence of covid-19 virus in the vaginal swab samples of female patients diagnosed with covid-19 and found radiologically compatible with viral pneumonia and to evaluate the risk of transmission of Covid-19 by sexual or vaginal delivery. In addition, it is necessary to evaluate the Covid-19 positivity in the vaginal samples taken again after treatment in patients with Covid-19 positivity in the vaginal swab.

B) Scientific Basis and Validity of Medical Research [In this section, indicate why the medical research is intended to be carried out, its basis and validity, by showing references related to the subject, not exceeding 300 words]:

There is a publication about the investigation of Covid-19 positivity in vaginal discharge including 10 patients (1). In this publication, no virus was detected in the vaginal swabs of female patients treated with Covid-19 pneumonia. A limited number of publications related to Covid-19 virus in pregnant women and sexual transmission have been published in the literature, and the need for new studies in this field has been stated in the reviews (2,3,6). In all cases with covid-19 disease in the pregnant population, it was observed in the 3rd trimester, which raised questions about the timing of delivery and delivery method (7). In a study of 35 sexually transmitted diseases, sexual transmission of this virus could not be demonstrated, but it was stated that more studies on this subject are needed due to the high number of asymptomatic patients and the high transmission rate of the virus (4). To evaluate the possibility of sexual transmission of this disease and the possibility of transmission to the newborn during vaginal delivery by examining the vaginal swabs of women who are not pregnant or pregnant, who are diagnosed with viral pneumonia due to Covid-19, and to prevent the spread of the disease and It will enable us to obtain important information to protect.

References

1.Lin Qiu, Xia Liu, Meng Xiao, Jing Xie, Wei Cao, Zhengyin Liu, Abraham Morse, Yuhua Xie, Taisheng Li, Lan Zhu, SARS-CoV-2 is not detectable in the vaginal fluid of women with severe COVID- 19 infection, Clinical Infectious Diseases,, ciaa375

2. Archives of Pathology & Laboratory Medicine Online. 2020. An Analysis Of 38 Pregnant Women With COVID-19, Their Newborn Infants, And Maternal-Fetal Transmission Of SARS-Cov-2: Maternal Coronavirus Infections And Pregnancy Outcomes. [online] Available at: <https://www.archivesofpathology.org/doi/abs/10.5858/arpa.2020-0901-SA> [Accessed 9 April 2020].

3.Chen, H., Guo, J., Wang, C., Luo, F., Yu, X., Zhang, W., Li, J., Zhao, D., Xu, D., Gong, Q ., Liao, J., Yang, H., Hou, W. and Zhang, Y., 2020. Clinical Characteristics And Intrauterine Vertical Transmission Potential Of COVID-19 Infection In Nine Pregnant Women: A Retrospective Review Of Medical Records.

4. Cui, P., Chen, Z., Wang, T., Dai, J., Zhang, J., Ding, T., Jiang, J., Liu, J., Zhang, C., Shan, W ., Wang, S., Rong, Y., Chang, J., Miao, X., Ma, X. and Wang, S., 2020.Clinical Features And Sexual Transmission Potential Of SARS-Cov-2 Infected Female Patients: A Descriptive Study In Wuhan, China.

5. Qiao, J., 2020. What Are The Risks Of COVID-19 Infection In Pregnant Women ?.

6. Schwartz, D. and Graham, A., 2020. Potential Maternal And Infant Outcomes From Coronavirus 2019-Ncov (SARS-Cov-2) Infecting Pregnant Women: Lessons From SARS, MERS, And Other Human Coronavirus Infections.

7. Taylor & Francis. 2020. Vertical Transmission Of Coronavirus Disease 19 (COVID-19) From Infected Pregnant Mothers To Neonates: A Review. [online] Available at: <https://www.tandfonline.com/doi/full/10.1080/15513815.2020.1747120> [Accessed 9 April 2020].

C) Method of the Research and Procedures to be Applied [In this section, features such as patient and healthy participant information, how and how often materials will be collected, parameters to be measured, should be specified in detail]:

In the study, female patients who were evaluated in the emergency service of Acıbadem Maslak Hospital and whose clinical findings were compatible with Covid-19 were diagnosed with viral pneumonia radiologically, were hospitalized in the service and vaginal Covid-19 will be investigated by taking a vaginal swab simultaneously with the nasopharyngeal swab before the treatment begins. Covid-19 swab samples will be studied in Acıbadem Central Laboratory. In patients whose nasopharyngeal swabs are covid-19 (+) and who are clinically and radiologically compatible with Sars-CoV2 disease will be investigated for vaginal covid-19 by taking vaginal swabs. Vaginal Covid-19 negativity will be re-evaluated by performing a vaginal Covid-19 test again after treatment in patients with positive vaginal Covid-19.

D) Inclusion and Exclusion Criteria of Volunteers [list by article]:

All female patients over the age of 18 who are clinically and radiologically considered to be covid-19 at the first application to the study will be included.

Patients who have previously received treatment for covid-19 will be excluded from the study.

E) Number of Volunteers to be Included in the Study:

60 voluntary patients who meet the criteria will be included in our research.

F) Duration of the Study:

The research is planned to be completed within 1 month.

G) Research Support [how and from where research expenditures will be covered, if any, institutions providing support and the amount of support]:

Due to the fact that Covid-19 tests are applied in certain centers and free of charge in our country, our research does not have an additional expenditure budget.

3. QUALIFICATION OF THE RESEARCH

3.1 Which one does your research fit (according to article 6 of the Directive) [Please tick]

A) Research within the scope of the Regulation on Clinical Research ()

() Drug Research

() Non-commercial Drug Research

() Research with Medical Devices

() Researches Using New Surgical Methods

() Drug Bioavailability and Bioequivalence Studies

(x) Investigation of Diagnosis and Screening Tests

B) Medical researches not covered by the Regulation on Clinical Research (X)

() Observational Drug Research

() Observational Epidemiological Studies

() Medical Research in the Form of Questionnaire, Questionnaire and Interview

(x) Medical Research Involving the Use of Human Biological Materials

(x) Research on Medical Records and Patient Information

() Qualitative Research

3.2 According to you, which risk assessment (according to article 7 of the Directive) your research fits [Please tick]

(X) Risk-Free Medical Research

() Medical Research with Minimal Risk *

() Medical Research Including Additional Risk *

() Medical Research Including Uncertain Risk

3.3 On which group of people your research will be conducted (according to Article 8 of the Directive) [Please tick]

() On Patients [With Treatment () Independent of Treatment (X)]

() On Healthy People

() On Vulnerable and Vulnerable Groups

() Babies and children

() Pregnant women, puerperant women and nursing mothers

() Those with limited learning ability

() Severe and chronic patients

() Emergency patients

() Patients in coma

() Intensive care patients

() Psychiatric patients

() Alzheimer's patients and patients with similar types of dementia

() Hopeless and helpless patients

( ) Prisoners

() Soldiers

() Students and medical staff

() People who have had an accident

() Persons subjected to violence

() Survivors

4. DISCLOSED CONSENT FORM [Prepare according to the nature of the research to cover the issues in article 29 of the Directive.

"Detection of coronavirus in the vaginal flora and evaluation of covid-19 vertical transmission risk in a patient diagnosed with Covid-19," conducted by Dr. Özgüç Takmaz. We invite you to research titled. The purpose of this study is to investigate whether the covid-19 virus detected in the respiratory system and throat is present in the vaginal flora. For this purpose, before your treatment, if you allow, we will investigate the presence of the covid-19 virus in vaginal secretions / discharge by taking a swab sample taken from your mouth and nasal cavity, which is a routine procedure for patients with suspected covid-19, from the vaginal area. The sampling to be made and its result will in no way affect the treatment to be given to you. No additional fee will be charged from you or your insurance company for these inspection procedures. In addition, the process of taking a vaginal swab will not harm the hymen in women who are not sexually active.

An estimated 59 other people will participate in the research besides you. Participation in this study is entirely voluntary. Reading and confirming this form will mean that you agree to participate in the research. However, you also have the right not to participate in the study or to stop working at any time after participation. The information obtained from this study will be used solely for research purposes and your personal information will be kept confidential; however, your data can be used for publication purposes. If you need more information about the purpose of the research now or later, you can ask the researcher now or contact the 'ozguc.takmaz@acibadem.com.tr' e-mail address and phone number 02123044497. When the research is completed, please forward it to the researcher if you want general / specific results to be shared with you.

Procedures to be Applied: After your hospitalization process is completed, a vaginal swab sample will be taken from you on the same day with the swab sample taken from your mouth and nasal cavity, which is routinely performed, before starting treatment. Your treatment will be planned according to the swab taken from your mouth and nasal cavity, and we can share the results of the swab made for research purposes with you afterwards. If your vaginal Covid-19 test is positive (yes) before treatment, your vaginal Covid-19 test will be repeated after treatment. With this study, we will investigate the sexual transmission of covid-19 disease and the risk of transmission during childbirth in the pregnant population. If you wish, we can send you a copy of the article when the research results are published.

I read the above information that should be given to the participant before the research and I understood the scope and purpose of the study I was asked to participate in, and my voluntary responsibilities. Written and verbal explanation about the study was made by the researcher / researchers named below. I was also verbally told about the possible risks and benefits of the study. Adequate confidence has been given that my personal information will be protected with care.

Under these circumstances, I agree to participate in the research in question of my own free will, without any pressure or suggestion.

Participant:

Name and surname:.............................................. .................................................. .....................................

Signature: e-mail: Telephone:

For Those Under Guardianship or Guardianship;

Parent or Guardian

Name and surname:.............................................. .................................................. .....................................

Signature:

Researcher's

Name-Surname: Dr. Özgüç Takmaz
