# Supplementary material for: Severe acute respiratory syndrome coronavirus (SARS-CoV-2) is not detected in the vagina: A prospective study
Source: PLoS One. 2021 Sep 30;16(9):e0253072. doi: 10.1371/journal.pone.0253072 (PMC8483336; doi:10.1371/journal.pone.0253072)
Supplement: Attachment — Submitted filename: Response to Reviewers.docx [file pone.0253072.s004.docx]

**Response to Reviewers and the Academic Editor**

To the Academic Editor,

We reviewed the reference list of our manuscript. Recently published a new article was discussed in the discussion section and added in the reference list (13^th^ article in the reference list). Additionally, an erratum of a referenced article was added in the reference list (6^th^ reference).

1- We revised the text for the journal requirements. And we are sure that our manuscript meets the PLOS ONE‘s style requirements in the revised manuscript.
2- Signed inform consents were obtained from all patients who recruited to the study. This information was added in the methods section.

**Reviewer #1**

1- How was the sample size of 60 arrived at?

Authors’ Response: We thank Reviewer #1 for asking this important statistical basis of our study. The sample size was 56 patients which was calculated on the basis of the positive vaginal swab Covid-19 test detection in 20% of Covid-19 patients with pneumonia, with 85% power at an α level of 0.05. The sample size was calculated in G*Power 3 (Heinrich-Heine-Universität Düsseldorf, Düsseldorf, Germany). Considering the drop out of some patients or samples, we planned to recruit 60 patients for our study. Sample size calculation was added in the methods section.

*“The sample size was 56 patients which was calculated on the basis of the positive vaginal SARS-CoV-2 test detection in 20% of Covid-19 patients with 85% power at an α level of 0.05.”*

2- English grammar needs attention. Use reported speech and check the few spelling mistakes. Do not start a sentence with a numeral or digit.

Authors’ Response: English grammar was checked and some spelling mistakes were corrected. Sentences which were started with numeral or digit were changed.

3- Inclusion criteria was 15-45 years. How come you have a 47 year-old woman?

Authors’ Response: We thank Reviewer #1 for this correction, 47 was misspelled in Table 1. Table 1 was corrected.

4- Refer to the Tables and Figures in the body of your paper especially under materials and Methods and the Results section. The Tables and Figures have information that must be explained in the text.

Authors’ Response: Table and Figure were referred in the material, methods and result sections. Information which was shown in the Table 1 and Figure 1 was explained and added in the Results section.

*“Descriptive analysis of patients’ characteristics and medical conditions were performed for 38 patients. (Figure1)”*

*“Of the thirty-eight patients, 27 had two or more symptoms.”*

*“Diabetes, hypothyroidism, chronic ischemic heart disease, lymphoma, congenital adrenal hyperplasia and Crohn disease were the other reported medical disorders. Five patients had more than one chronic medical condition.”*

5- Make it clear that the descriptive analysis of symptoms, medical conditions and characteristics of patients were based on the 38 women who had the vaginal swabs tested.

Authors’ Response: Thank you for this important point. We added in the results section that the descriptive data and vaginal swab of 38 patients analyzed in the study. Also, number of analyzed patients were added in the Table 1.

*“Descriptive analysis of patients’ characteristics and medical conditions were performed for 38 patients. (Figure1)”*

6- Explain the methods used to determine or assess the symptoms, medical conditions and BMI under materials and methods.

Authors’ Response: Symptoms, chronic medical conditions and the patient characteristics were determined by the examination performed by the infectious disease physician on the first day of their hospitalization. This information was added in the Materials and Methods section.

“*Symptoms and the chronic medical conditions of the patients were determined by examination performed by the attending physician of the infectious disease department. The data of the chronic medical conditions, symptoms and the patients’ characteristics (age, BMI) were recorded from the inpatient charts of the patients, which was filled by the infectious disease physician on the first day of the hospitalization.”*

7- Make it clear if patients had more than one symptom or medical condition.

Authors’ Response: Thank you for this important point. Twenty-seven patients had more than one symptoms and 5 patients had more than one chronic conditions. This information was added in the results section.

*“Of the thirty-eight patients, 27 had two or more symptoms.”*

*“Five patients had more than one chronic medical condition.”*

8- Would it have been better to do all the 60 women in the sample considering that it may have been a minimum risk procedure.
What was the study design? You mentioned vaginal control swabs under methods. What were these and where are they reported?

Authors’ Response: Before the study was started, sample size of 56 patients was calculated for reaching a 85% power, as we stated in the first answer of the letter. Considering both the negative results of vaginal samples of the first 38 patients in our study and also the negative results of other small case series reported, the authors decided to close the patient enrollment. We think the negative vaginal swab results of 38 patients would provide sufficient information on the subject. Further studies designed with a different methodology may bring new information on the subject.

In our study design, control vaginal swabs was planned to obtain from the patients who had positive test result for vaginal SARS-CoV-2 swab test. Because of the none of the patients had positive vaginal swab test for SARS-CoV-2, we did not obtain any control vaginal swab test.

9- In Table 1, Anosmia should be 24% instead of the stated 23%.

Authors’ Response: The percentage of patients with anosmia symptoms was changed to 24% in Table 1.

10- Indicate the number of patients analysed in the Table 1.

Authors’ Response: Number of patients analyzed (n=38) were added in the Table 1.

11- There is no Mean and standard deviation shown in Table 1

Authors’ Response: Thank you for this point. We removed ‘Mean and standard deviation’ from the Table 1.

**Reviewer #2**

1- Please change the term “colonization” to “infection” as it is more accurate (e.g., viruses infect, bacteria colonize).

Authors’ Response: The term ‘colonization’ was changed to ‘infection’ in the introduction section.

2- Although the authors highlight that there is not much known about this area, the should include an additional paragraph about incidence of viral transmission from mom to baby. Recent reports of interest include below…

Authors’ Response: Thank you for this important contribution to our manuscript. We added a paragraph in the introduction section for vertical transmission of SARS-CoV-2. Also, new references for the paragraph have been added to the references.

*“Additionally, another important unknown aspect of the SARS-CoV-2 is the possibility of fetal transmission. Many studies reported that SARS-CoV-2 PCR tests were negative for newborns [13, 14]. However, some of them found IgM antibody positive in newborn blood sample which can be a sign for fetal infection [8, 15]. Some studies have detected the SARS-CoV-2 in newborns, although the frequency is very low [16, 17]. Recently published a meta-analysis reported that vertical transmission risk cannot be excluded with the existing literature. However, if vertical infection is possible, this probability is very low [18]. “*

3- Please modify the following statement for clarity. “Patients with positive test results were planned to obtain control vaginal swabs”. Do the authors mean that patients with positive nasal swab tests were intended to undergo vaginal swabs?

Authors’ Response: We thank Reviewer #2 for allowing us to clarify our study design. The sentence was changed to *“A control vaginal swab PCR test was planned for patients whose vaginal swab PCR test was positive.”*

4- Similarly, it is not clear what the authors mean in the subsequent sentence (“Owning to the fact that the test results of the first 38 patients were negative, the study was finished earlier”). Is the sentence meant to convey that the first 38 patients that tested positive by nasal swab PCR had negative vaginal swabs for SARS-CoV-2, and therefore the study was terminated early? Please clarify.

Authors’ Response: We thank Reviewer #2 for his/her contribution to the explanation of our study methodology. The study was terminated early, because of the negative vaginal swab PCR test results of the first 38 patients. And the sentences was changed to “ *the first 38 patients who tested positive by nasal swab PCR had negative vaginal swabs for SARS-CoV-2, and therefore the study was terminated early”*

5- In Table 1, the column numbers for medical disorder add to equal 43. Is this correct as only 41 vaginal swabs were obtained? If so, then can the authors please provide a note in the table legend that one or two patients had more than one medical condition?

Authors’ Response: In the study results, the data of 38 patients was analyzed. 22 patients had no chronic medical condition, five patients had more than one medical condition. We added in the legend of Table 1 that five patients had more than one medical condition.

6- Suggest using terms like detected, transmitted, infected instead of “colonized”.

Authors’ Response: The term ‘colonized’ was changed to ‘detected’ or ‘infected’ in the manuscript.

7- Please also consider the references suggested in the introduction as it relates to the discussion.

Authors’ Response: We added a paragraph about the vertical transmission risk of the SARS-CoV-2 in the discussion section.

*“Other important issue for SARS-COV-2 is the detecting the vertical infection risk. The absence of SARS-CoV-2 in the vagina does not mean that vertical infection cannot occur. In a meta-analysis conducted on this subject and including 29 studies, it was reported that SARS-COV-2 can cause 3.2% vertical infection. However, the evidence for the transmission of SARS-COv-2 to the fetus during the intrauterine period is quite limited.”*

*8-* The authors may wish to indicate in the Figure 1 legend that 41 swabs were collected but only 38 were assessed due to technical limitations.

Authors’ Response: The sentence ‘41 swabs were collected but only 38 were assessed due to technical limitations’ was added in the Figure 1 legend.

**Reviewer #3**

1- State and justify the study’s target sample size with a pre-study statistical power calculation. Typically a power calculation for a study such as this would be based on the anticipated proportion of women whose swabs test positive for COVID and the corresponding 95% confidence interval.

Authors’ Response: We thank Reviewer #3 for asking this important statistical basis of our study. The sample size was 56 patients which was calculated on the basis of the positive vaginal swab SARS-CoV-2 test detection in 20% of Covid-19 patients with 85% power at an α level of 0.05. The sample size was calculated in G*Power 3 (Heinrich-Heine-Universität Düsseldorf, Düsseldorf, Germany). Considering the drop out of some patients or samples, we planned to recruit 60 patients for our study. Sample size calculation was added in the methods section.

*“The sample size was 56 patients which was calculated on the basis of the positive vaginal SARS-CoV-2 test detection in 20% of Covid-19 patients with 85% power at an α level of 0.05.”*
