# Supplementary material for: Severe acute respiratory syndrome coronavirus (SARS-CoV-2) is not detected in the vagina: A prospective study
Source: PLoS One. 2021 Sep 30;16(9):e0253072. doi: 10.1371/journal.pone.0253072 (PMC8483336; doi:10.1371/journal.pone.0253072)
Supplement: Attachment — Submitted filename: Response to Reviewers .docx [file pone.0253072.s005.docx]

**Response to Reviewers and the Academic Editor**

To the Academic Editor,

First of all, all authors thank Academic Editor and Reviewers for their positive contributions to our manuscript. In the second revision, the manuscript underwent English editing by a native speaker. And all the concerns which were pointed out by the first Reviewer were addressed.

**Reviewer #1**

**1- The paper needs to be edited for standard English grammar.**

Authors’ Response: The manuscript was edited for English grammar by a native speaker who is experienced in grammar editing. After grammar editing, the manuscript became more fluent. Thank you for this contribution to our manuscript.

**2- The Mean and Standard deviation appears under methods but was not used in the analysis of data. please remove this and explain or use the methods for analysis that were done.**

Authors’ Response: Thank you for this clarification of our statistical methodology. Characteristics of the patients (BMI and age) did not distribute normally in our study. The Mann-Whitney U test was used to evaluate non-normally distributed data, which were reported as median (interquartile range, minimum, maximum). Mean and standard deviation were not used for presenting the patient characteristics. We removed this statement from our statistical methodology and we added the tests that we used for analyze.

‘’ The assumption of normality was made by Shapiro-Wilk test. As the characteristics of the patients distributed non-normally, the Mann-Whitney U test was used and the results were given as median with interquartile range.’’

**3- Under results section correct 'swap'to 'swab' and 'chronicle'' to 'chronic'.**

Authors’ Response: We corrected the ‘swap’ to ‘swab’ and ‘chronicle’ to ‘chronic’ in result section.

**4- You had mentioned that 47 years was an error and removed as it was not part of the inclusion criteria. However this still appears in the under the results section. Did you actually include a 47 year old patient?**

Authors’ Response: Authors thank Reviewer 1 for this important correction. Before the writing process of our manuscript, The Table 1 was created. In the Table 1, it was misspelled, then result section was written on the basis of the Table. In the first round of revision, we checked the ages of all patients of the study. And none of the patients was older than 45 years old. We corrected the result section for this misspelling.

**5- Regarding the positive vaginal control swab which you earlier clarified. For observational studies like the current one a control is one that does not possess the condition or disease and a case is one with the condition or disease. then in the analysis you check if the cases are associated with certain risk factors like in your case, age, BMI, medical conditions, symptoms etc. In your study, the control was taken as one with the condition or disease. please clarify.**

Authors’ Response: Our study did not have any control group that did not possess SARS-CoV-2 pneumonia. In our study design, if the vaginal swab test was positive for SARS-CoV-2, a control (repeat) vaginal swab test would have been performed later in the follow up period. These patients would not be control group. However, none of the patients’ vaginal swab test was positive, therefore we did not have any repeat (control) vaginal test for any patients. If the Reviewer 1 wants to change the word ‘control’, we will change or remove it from the manuscript.
